# Angiotensin-converting enzyme 2 (ACE2) polymorphisms and susceptibility of severe SARS-CoV-2 in a subset of Pakistani population

**DOI:** 10.1186/s12985-023-02091-2

**Published:** 2023-06-12

**Authors:** Santosh Kumar Sidhwani, Talat Mirza, Ambrina Khatoon, Fouzia Shaikh, Rizma Khan, Omer Ahmed Shaikh, Abdulqadir J. Nashwan

**Affiliations:** 1 Department of Pathology, Ziauddin University, Karachi, Pakistan; 2 Department of Research, Ziauddin University, Karachi, Pakistan; 3 Department of Molecular Medicine, Ziauddin University, Karachi, Pakistan; 4 Department of Molecular Genetics, Ziauddin University, Karachi, Pakistan; 5 Department of Medicine, Ziauddin University, Karachi, Pakistan; 6 Nursing for Education Hamad Medical Corporation, P.O. Box 3050, Doha, Qatar

**Keywords:** SARS-CoV-2, ACE2, Polymorphisms, rs2285666, Variants, SNPs

## Abstract

Science is digging for the varied presentation of COVID-19 patients exposed to the same risk factors, and medical conditions may be influenced by the presence of polymorphic genetic variants. This study investigated the link between ACE2 gene polymorphisms and the severity of SARS-CoV-2. This cross-sectional study recruited COVID-19 PCR-positive patients by consecutive sampling from Ziauddin Hospital from April to September 2020. DNA was extracted from whole blood, followed by gene amplification and Sanger’s sequencing. Most of the patients, 77: 53.8%, were serious. Males were higher (80; 55.9%) with age more than 50 years (106: 74.1%). We found 22 ACE2 SNPs. rs2285666 SNP was most prevalent with 49.2% CC, 45.2% TT, 4.8% CT heterozygosity, and 0.8% AA genotypes. Variants with multiple genotypes were also insignificantly associated with the severity of COVID-19 in the analysis of the dominant model. Only rs2285666 had a significant statistical link with gender (p-value 0.034, OR; 1.438, CI; 1.028–2.011) while rs768883316 with age groups (p-value 0.026, OR; 1.953, CI; 1.085–3.514). Haplotypes ATC of three polymorphisms (rs560997634, rs201159862, and rs751170930) commonly found in 120 (69.77%) and TTTGTAGTTAGTA haplotype consisting of 13 polymorphisms (rs756737634, rs146991645, rs1601703288, rs1927830489, rs1927831624, rs764947941, rs752242172, rs73195521, rs781378335, rs756597390, rs780478736, rs148006212, rs768583671) in 112 (90.32%) had statistically significant association with the severity having p = value 0.029 and 0.001 respectively. Males of old age and diabetics are found to have more severe COVID-19 infection in the current study. We also found that common ACE2 polymorphism rs2285666 influences the susceptibility of acquiring the severe SARS-CoV-2 infection.

**Supplementary Information:**

The online version contains supplementary material available at 10.1186/s12985-023-02091-2.

## Introduction

The SARS-CoV-2 virus infects some patients severely and fatally, primarily, but not solely, older people with serious underlying medical conditions [1]. One of the most reliable indicators of mortality is hypertension. Angiotensin-converting enzyme inhibitors or angiotensin II receptor blockers may help the first stage of viral infection because SARS-CoV-2 needs the ACE2 protein to permeate the cell membrane and because hypertension people have a higher incidence of sequelae [2]. However, possibly the inflammatory stage of the disease would benefit from these same medications [3].

Blocking ACE2 has been proposed by several authors as a viable method to lower the viral SARS-CoV-2 burden in the pneumocytes and stop the virus from spreading to other organs [4]. In contrast, blocking ACE2 in COVID-19 individuals who are already infected could be harmful because it would lead to less angiotensin 1–7 synthesis, which has been shown to have anti-inflammatory and antifibrotic properties through its receptor [5]. Humans infected with the SARS-Cov-2 virus develop a very varied illness with unpredictably high mortality rates. Some people experience no symptoms at all, while others experience distress, microvascular thrombosis, multi-organ failure, and death as a result of a chain of infections and inflammatory processes. Although prognostic indicators have been established, there is still a lot of unexplained variation [6].

It is likely that changes in the activity of receptor-specific proteins, influenced by the presence of many polymorphic genetic variants in the population, contribute to increased susceptibility to infection or enhanced viral replication efficiency. The existence of multiple polymorphisms in the ACE (I/D), ACE2 (rs2074192, rs1978124, rs2074809, rs2074666) genes could explain both the tendency to infection, the expansion to different organs, and the severity of COVID-19 clinical manifestations [7]. Therefore, we investigated the scientific links between ACE2 gene polymorphisms and the severity of COVID-19.

## Methodology

### Patient recruitment and specimen collection

This cross-sectional study was conducted following the approval of the research and ethics review committees of Ziauddin University (Reference code: 2650920SKPAT) as per institutional guidelines. A total of 143 PCR-positive patients presented to COVID-19 outpatient and inpatient departments and the intensive care unit (ICU) at Ziauddin Hospital Clifton, KDLB, and North Nazimabad sites from April 2020 to September 2020, using the non-probability consecutive sampling technique were included. Written informed consent was obtained from the patients or from the guardian of each participant under 18 years of age. Patients under 14 years of age, mentally challenged, patients, post chemotherapy and radiotherapy, and patients with any malignant condition were excluded.

SARS-CoV-2 RNA positivity was determined using qualitative RT-PCR with in vitro diagnostic kits, following the manufacturer’s protocol. The electronic patient records provided demographics, clinical features, and laboratory results. The age, gender, medical history including early symptoms like fever, cough, dyspnea, and outcome of each patient were recorded. According to the CDC’s standard grades of severity were defined as ‘Asymptomatic’ with no signs or symptoms; ‘Mild’ referred to patients with no dyspnea; whether outpatient or inpatients; ‘Moderate’ referred to in-patients admitted to a ward or ICU who showed the signs of dyspnea but did not need oxygen; ‘Severe’ labeled as hospitalized patients who required high flow oxygen; ‘Critical’ considered all patients who required mechanical ventilation or all COVID-19-related deaths that occurred during the hospital stay or both [8]. To assess statistical risk, the study subjects were divided into two categories: serious and non-serious patients. The non-serious group included asymptomatic, mild, and moderate cases, while the serious group consisted of all severe and critical patients who were admitted to the hospital, whether inwards or in the ICU.

### DNA extraction, quantification

The DNA was extracted from the entire blood using the Qiagen Kit (QIAamp DNA mini blood Kit, Cat. No. 51306) in accordance with the manufacturer’s guidelines. The concentration of each DNA sample was verified using a Multiskan Sky spectrophotometer, and purity was determined by calculating the A260/280 ratio. Gel electrophoresis was performed to assess DNA integrity.

The NCBI’s Thousand Genome Database offers over 1700 ACE2 gene polymorphism variants across diverse populations worldwide. As of August 2020, 124 of these variants have been observed in the East Asian population, including Pakistan, which shares a similar genetic makeup to our population. Notably, six frequent variants (rs201159862, rs1601703288, rs781378335, rs752242172, rs2285666, and rs768883316) have been identified [9].

### Polymerase chain reaction (PCR)

The primers were designed against the more frequent variants correlated with the COVID-19 in East Asian population. Primers for ACE2 were obtained commercially (Macrogen, peniconpk, 25 nmol), forward primer, 5’-TCATGTCCTTGCCCTTATAGTTCC-3’ and reverse primer 5’-CTATACTACCGCATCACTTTTTGGT-3’. The PCR cycling was performed as initial denaturation at 94ºC for 5 min followed by 35 cycles of 1 min at 94ºC, 1 min at 58.5ºC and 1 min at 72ºC with a final extension of 5 min at 72ºC. (SimpliAmp, Thermal Cycler, applied biosystems, Thermo Fisher Scientific, Ref: A24812, SN: 228,007,070) Amplification products were observed using submerged 2% agarose gel electrophoresis. (Supplementary Fig. 1)

### Sanger’s sequencing and bioinformatic analysis

Sanger’s sequencing was used to analyze all the ACE2 products (Size 800 bp). However, 19 samples had missed reads and noise and were subsequently filtered out of the analysis. This left 126 samples for genetic association and statistical analysis. The sequencing procedure was outsourced and performed with Lab Genetix in Lahore. MEGA X software was used for sequence alignment and trimming. The datasets generated and analyzed during the study are available at NCBI, GenBank repository, Submission# BankIt2673536 ZU15 OQ443069, https://www.ncbi.nlm.nih.gov/WebSub/.

### Statistical evaluation

SPSS version 21 was used for all statistical evaluations conducted on COVID-19. Categorical variables were subjected to frequency calculations, while quantitative variables were analyzed using percentage calculations. Fisher’s exact/Chi-square testing was employed to determine the correlation between COVID-19 severity and clinicopathological features. The Hardy-Weinberg equilibrium (HWE) for genotype distributions was computed using the chi-square (χ2) test in Haploview. Regression analysis was utilized to calculate the odds ratio (OR) at a 95% confidence interval (CI) to assess the association between genotype and allele frequencies, as well as other variables, and the likelihood of COVID-19 severity. A P-value less than 0.05 was considered significant for all estimates, which had a two-sided distribution.

## Results

### Demographic and clinical characteristics of the patients

Out of all patients who tested positive for PCR, 77 of them (53.8%) had serious cases while 66 (46.2%) had non-serious cases. More than half of the patients were males (80, 55.9%) and were over 50 years old (106, 74.1%) when they first presented with symptoms. The majority of patients had diabetes mellitus (71, 49.7%) and hypertension (83, 58%), while some also had other known diseases such as cardiovascular disease (22, 15.4%) or respiratory disease (7, 4.9%). It should be noted that cardiovascular disease in this context refers to ischemic heart disease, coronary artery disease, and valvular disease, but not arterial diseases or hypertension. Endocrine diseases do not include diabetes mellitus. Fever (90, 62.9%) was the most common symptom reported by patients, followed by dyspnea (87, 60.8%). Almost all patients had raised levels of inflammatory markers, with procalcitonin (139, 97.2%) being the most frequently elevated marker.

After analyzing demographic and clinical characteristics in relation to COVID-19 severity, we found a significant statistical association between several factors. Fever (p-value, 0.001), cough (p-value, 0.004), dyspnea (p-value, 0.001), loss of taste (p-value, 0.043), de-dimer (p-value, < 0.001), LDH (p-value, < 0.001), CRP (p-value, < 0.001), and outcome (p-value, < 0.001) all showed significant correlations. Fever, cough, and dyspnea were present in all cases ranging from mild to critical severity, while loss of taste and smell were more common in mild to moderate cases. Please refer to Table 1 for more information.

**Table 1:** Statistical association of demographic and clinical characteristics with severity of COVID-19: Fever, cough, dyspnea, loss of taste, elevated d-dimer, LDH, and CRP levels were all significantly associated with the severity of the disease. Fever, cough, and dyspnea were commonly present in all severity levels, while loss of taste and smell were more frequently associated with mild and moderate cases

Characteristics	n = 143	Severity		P value^a^
		**Serious** **77 (53.8)**	**Non-Serious** **66 (46.2)**	
Age (Years) <=50 > 50	37 (25.9) 106 (74.1)	18 (12.6) 59 (41.3)	19 (13.3) 47 (32.9)	0.566
Gender Male Female	80 (55.9) 63 (44.1)	44 (30.8) 33 (23.1)	36 (25.2) 30 (21)	0.866
Ethnicity Sindhi Urdu Punjabi Pathan	35 (24.5) 82 (57.3) 18 (12.6) 8 (5.6)	15 (10.5) 45 (31.5) 14 (9.8) 3 (2.1)	20 (14) 37 (25.9) 4 (2.8) 5 (3.5)	0.081
Hypertension Yes No	83 (58) 60 (42)	46 (32.2) 31 (21.7)	37 (25.9) 29 (20.3)	0.735
Diabetes Mellitus Yes No	71 (49.7) 72 (50.3)	42 (29.4) 35 (24.5)	29 (20.3) 37 (25.9)	0.242
Fever Yes No	90 (62.9) 53 (37.1)	59 (41.3) 18 (12.6)	31 (21.7) 35 (24.5)	0.001^*^
Cough Yes No	58 (40.6) 85 (59.4)	40 (28) 37 (25.9)	18 (12.6) 48 (33.6)	0.004^*^
Dyspnea Yes No	87 (60.8) 56 (39.2)	64 (44.8) 13 (9.1)	23 (16.1) 43 (30.1)	0.001^*^
Arthralgia Yes No	18 (12.6) 125 (87.4)	9 (6.3) 68 (47.6)	9 (6.3) 57 (39.9)	0.803
Ageusia Yes No	4 (2.8) 139 (97.2)	0 (0) 77 (53.8)	4 (2.8) 62 (43.4)	0.043^*^
Anosmia Yes No	6 (4.2) 137 (95.8)	2 (1.4) 75 (52.4)	4 (2.8) 62 (43.3)	0.415
TLC Raised Normal Below Normal	120 (83.9) 21 (14.7) 2 (1.4)	62 (43.3) 15 (10.5) 0 (0)	58 (40.6) 6 (4.2) 2 (1.4)	0.075
De-Dimer Raised Normal	105 (73.4) 38 (26.6)	66 (46.2) 11 (7.7)	39 (27.3) 27 (18.9)	< 0.001^*^
Ferritin Raised Normal	100 (69.9) 43 (30.1)	56 (39.2) 21 (14.7)	44 (30.8) 22 (15.4)	0.468
LDH Raised Normal	127 (88.8) 16 (11.2)	76 (53.1) 1 (0.7)	51 (35.7) 15 (10.5)	< 0.001^*^
CRP Raised Normal	92 (64.3) 51 (35.7)	62 (43.3) 15 (10.5)	30 (21) 36 (25.2	< 0.001^*^
Pro-calcitonin Raised Normal	139 (97.2) 4 (2.8)	74 (51.7) 1 (0.7)	65 (45.5) 3 (2.1)	0.642
Outcome Discharged Deaths	110 (76.9) 33 (23.1)	48 (33.6) 29 (20.3)	62 (43.4) 4 (2.8)	< 0.001^*^

Categorical data represented as n and frequency in percentages (%), *significant p value (*P* < 0.05), ^a^Chi square test/Fischer’s Exact Test, TLC: Total Leukocyte Count, LDH: Lactate Dehydrogenase, CRP: C-Reactive Protein

### Association of ACE2 variants and the severity of the disease

**Fig. 1:**
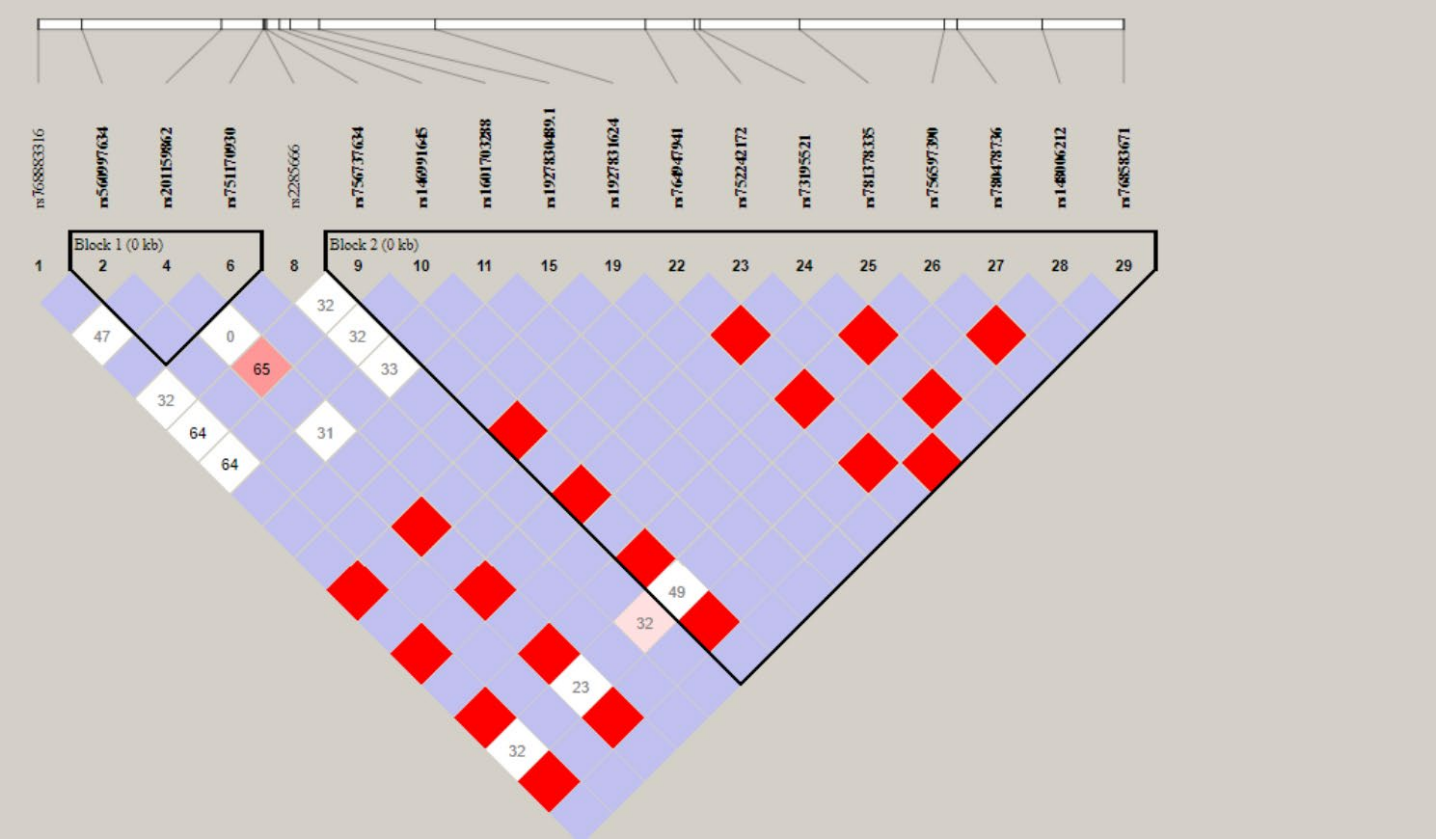
Haploview result belonging to block 1 of ACE2 polymorphisms, contains (rs560997634, rs201159862, and rs751170930) and block 2 that contains (rs756737634, rs146991645, rs1601703288, rs1927830489, rs1927821624, rs764947491, rs752242172, rs73195521, rs781378335, rs756597390, rs780478736, rs148006212 and rs768583671) according to genotyping data of this study. The red color means 100% of linkage disequilibrium (D’ = 1). Boxes number referred to linkage disequilibrium (D’) between SNPs, boxes with no number mean 100% linkage (D’ = 1). Color legend: i) Bright red = high D’; White = low D’; iii) Purple = High D’ but low LOD score (see Haploview documentation for further details; http://www.broad.mit.edu/mpg/haploview

**Fig. 2:**
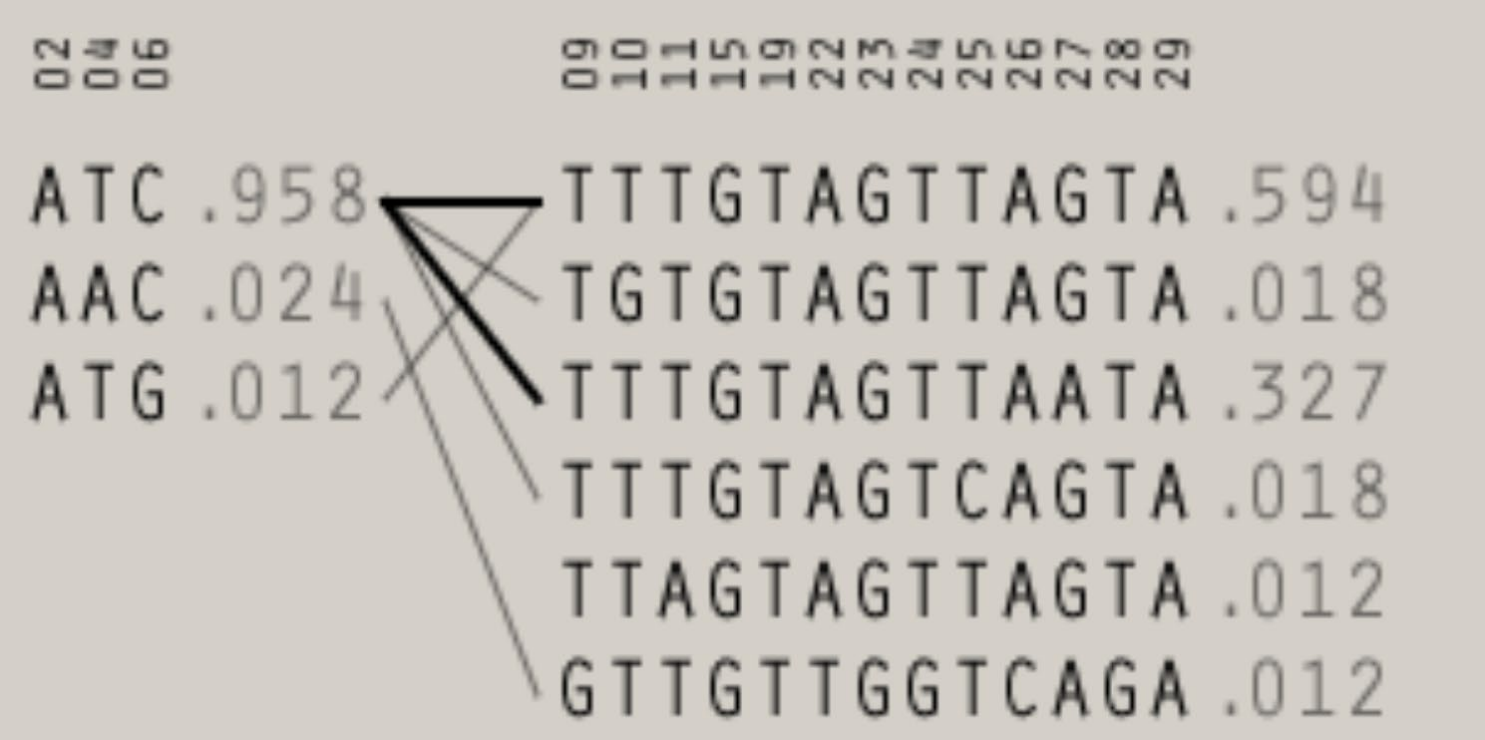
Haplotypes and haplotype frequencies showing association among each other: The bold lines display a favorable correlation between the ATC haplotypes observed in 120 patients, out of which 66 were severely affected, and the presence of TTTGTAGTTAGTA and TTTGTAGTTAATA haplotypes in 112 patients, out of which 64 were also severely affected. It is important to note that the impact of these haplotypes varies across different populations, depending on environmental factors

In our recent survey, we discovered 29 ACE2 variants within a specific range of base pairs (position: 15,592,100 to 15,592,699). Out of these, 7 variants had no changes in their sequences while the remaining 22 ACE2 variants had altered genotypes. Among these, 15 variants were intronic, 4 were splice donor region variants, 2 were missense, and one was a synonymous variant. Additionally, two variants had insertion/deletion and one had insertion. Due to the low number of minor homozygotes (MAF < 0.1, Table: 2), we only analyzed these polymorphisms using a dominant model. We used these associations for further analyses and considered the linkage disequilibrium between gene polymorphisms (Figures: 1 and 2). To control the expected proportion of false positives (False Discovery Rate [FDR]) instead of the more stringent Bonferroni correction, we conducted an exploratory selection of associations. Therefore, we used associations with FDR < 0.1 in haplotype analyses. Firstly, we generated haplotype blocks using the algorithm of four-gamete rules observed at a frequency > 0.01. Then, we tested if the observed frequencies of haplotypes deviated from expected under linkage equilibrium for each block. Finally, we assessed the association between haplotypes and phenotypes using a permutation procedure (Supplementary Table: 1).

**Table 2:** Minor allele frequency (MAF) and results of exact test to assess deviations from Hardy-Weinberg equilibrium (HWE)

*ACE2*	Major Allele	Minor Allele	Class	Consequences	MAF	HWE
rs768883316	T	A	SNP	Intronic Variant	0.055	2.6928E-6
rs560997634	A	C	SNP	Intronic Variant	0.006	1.00
rs201159862	T	A	SNP	Intronic Variant	0.024	0.0206
rs751170930	C	G	Insertion	Splice donor region variant	0.012	0.0206
rs1569241829	C	G	SNP	Splice donor 5th base variant	0.012	0.0206
rs2285666	T	G	SNP	Splice donor region variant	0.497	8.2762E-15
rs756737634	T	G	Indel	Splice donor region variant	0.018	0.0206
rs146991645	T	G	SNP	Synonymous variant	0.018	0.0206
rs1601703288	T	A	SNP	Missense variant	0.018	1.00
rs1391451327	A	T	SNP	Missense variant	0.055	3.5006E-5
rs1927830489	G	T	SNP	Intronic Variant	0.006	1.00
rs1927831624	T	C	SNP	Intronic Variant	0.006	1.00
rs764947941	A	T	SNP	Intronic Variant	0.012	0.0206
rs752242172	G	T	SNP	Intronic Variant	0.006	1.00
rs73195521	G	T	SNP	Intronic Variant	0.006	0.0206
rs781378335	T	C	SNP	Intronic Variant	0.018	0.0206
rs756597390	A	C	SNP	Intronic Variant	0.012	0.0206
rs780478736	G	A	SNP	Intronic Variant	0.345	2.8697E-14
rs148006212	T	G	SNP	Intronic Variant	0.012	0.0206
rs768583671	A	C	Indel	Intronic variant	0.006	1.00

Altered variants had the MAF of < 0.1. Out of all 4 variants were splice donor site, missense, and insertion. rs560997634, rs1601703288 and, rs768583671 were in linkage equilibrium (LE). HWE = 1 considered as variants are in LE

In COVID-19-positive patients, the most common variant observed was rs2285666. Out of these patients, 49.2% had the CC genotype, 45.2% had the TT genotype, 4.8% had the CT heterozygosity, and 0.8% were genotypic carriers of AA. The second most frequent SNP was rs1391451327, with 93.5% of patients having the AA genotype, 5.7% having the TT genotype, and 0.8% having the GG genotype. Another variant, rs781378335, had a genotypic frequency of 97.6% TT and 2.4% CC. Although these SNPs do not have a significant association with the severity of the disease, changes were observed in more severe and critical patients in the ICU. This suggests that these variants may increase susceptibility to acquiring severe or critical COVID-19 infections. Some genotypic variants of ACE2 were also found to be associated with mild and moderate cases. However, variants with multiple genotyping were not found to be associated with COVID-19 severity in the analysis of the dominant model. Please refer to Tables 3 and 4 for more information.

**Table 3:** Genetic association study of ACE2 variants with severity of COVID-19. rs2285666 was a commonly altered variant (63 patients) followed by rs139141327 (8 patients). None of the variants had a statistically significant association with the severity of COVID-19 though the variants were found in the serious groups. ^b^Benjamini-Hockberg method. CI, confidence Interval; FDR, false discovery rate; NA, does not apply; OR, odds ratio

ACE2 Single nucleotide variant	All, n = 124	Severity		P	OR (CI 95%)	FDR^b^
		**Serious** **n = 68**	**Non-Serious** **n = 56**			
rs180878567 Reference (AA) Altered (TT) Altered (GG)	121 (0.976) 2 (0.016) 1 (0.008)	68 (1.00) 0 (0) 0 (0)	53 (0.946) 2 (0.036) 1 (0.018)	0.999	NA	1.998
rs751227277 Reference (GG) Altered (TT)	123 (0.992) 1 (0.008)	67 (0.985) 1 (0.015)	56 (1.00) 0 (0)	1.000	NA	1.571
rs1391451327 Reference (AA) Altered (GG) Altered (TT)	116 (0.935) 1 (0.008) 7 (0.057)	61 (0.897) 1 (0.015) 6 (0.088)	55 (0.982) 0 (0) 1 (0.018)	0.096	0.538 (0.260–1.116)	2.112
rs1469916451 Reference (TT) Altered (CC)	122 (0.984) 2 (0.016)	67 (0.985) 1 (0.015)	55 (0.982) 1 (0.018)	0.890	0.906 (0.224–3.664)	2.797
rs1601703288 Reference (TT) Altered (AA)	121 (0.976) 3 (0.024)	67 (0.985) 1 (0.015)	54 (0.964) 2 (0.036)	0.463	0.739 (0.329–1.659)	5.093
rs201159862 Reference (TT) Altered (AA)	121 (0.976) 3 (0.024)	67 (0.985) 1 (0.015)	54 (0.964) 2 (0.036)	0.463	0.739 (0.329–1.659)	3.395
rs751170930 Reference (CC) Altered (GG)	123 (0.992) 1 (0.008)	67 (0.985) 1 (0.015)	56 (1.00) 0 (0)	1.000	NA	1.466
rs756737634 Reference (TT) Altered (GG)	122 (0.984) 2 (0.016)	67 (0.985) 1 (0.015)	55 (0.982) 1 (0.018)	0.890	0.821 (0.50-13.427)	2.447
rs1927831624 Reference (TT) Altered (CC)	123 (0.992) 1 (0.008)	67 (0.985) 1 (0.015)	56 (1.00) 0 (0)	1.000	NA	1.375
rs764947941 Reference (AA) Altered (TT)	123 (0.992) 1 (0.008)	67 (0.985) 1 (0.015)	56 (1.00) 0 (0)	1.000	NA	1.294
rs73195521 Reference (TT) Altered (GG)	123 (0.992) 1 (0.008)	67 (0.985) 1 (0.015)	56 (1.00) 0 (0)	1.000	NA	1.222
rs752242172 Reference (GG) Altered (TT)	122 (0.984) 2 (0.016)	67 (0.985) 1 (0.015)	55 (0.982) 1 (0.018)	1.000	NA	1.157
rs781378335 Reference (TT) Altered (CC)	121 (0.976) 3 (0.024)	68 (1.00) 0 (0)	53 (0.946) 3 (0.054)	0.999	NA	1.831
rs756597390 Reference (AA) Altered (CC) Altered (TT)	122 (0.984) 1 (0.008) 1 (0.008)	67 (0.985) 0 (0) 1 (0.015)	55 (0.982) 1 (0.018) 0 (0)	0.622	0.686 (0.154–3.062)	2.736
rs148006212 Reference (TT) Altered (GG)	123 (0.992) 1 (0.008)	67 (0.985) 1 (0.015)	56 (1.00) 0 (0)	1.000	NA	1.100
rs780478736 Reference (GG) Altered (AA)	121 (0.976) 3 (0.024)	67 (0.985) 1 (0.015)	54 (0.964) 2 (0.036)	0.463	0.635 (0.189–2.136)	2.546
rs768583671 Reference (AA) Altered (CC) Altered (GG) Altered (TT)	121 (0.976) 1 (0.008) 1 (0.008) 1 (0.008)	65 (0.955) 1 (0.015) 1 (0.015) 1 (0.015)	56 (1.00) 0 (0) 0 (0) 0 (0)	0.999	NA	1.1.690
rs2285666 Reference (CC) Altered (AA) Altered (CT) Altered (TT)	61 (0.492) 1 (0.008) 6 (0.048) 56 (0.452)	32 (0.48) 1 (0.015) 2 (0.029) 33 (0.485)	29 (0.51) 0 (0) 4 (0.07) 23 (0.42)	0.966	0.993 (0.716–1.377)	2.125
rs1569241829 Reference (CC) Altered (GG)	123 (0.992) 1 (0.008)	67 (0.985) 1 (0.015)	56 (1.00) 0 (0)	1.000	NA	1.047
rs768883316 Reference (TT) Altered (CC) Altered (AA)	117 (0.944) 2 (0.016) 5 (0.04)	64 (0.942) 0 (0) 4 (0.058)	53 (0.946) 2 (0.036) 1 (0.018)	0.655	1.140 (0.642–2.023)	2.401
rs560997634 Reference (AA) Altered (CC)	122 (0.984) 2 (0.016)	67 (0.985) 1 (0.015)	55 (0.982) 1 (0018)	0.890	1.218 (0.074–19.92)	2.175
rs1927830489 Reference (GG) Altered (TT)	123 (0.992) 1 (0.008)	68 (1.00) 0 (0)	55 (0.0982) 1 (0.018)	1.000	NA	1.00

**Table 4:** Dominant model analyses for ACE2 genetic variants with multiple genotypes. Six variants listed have the multiple altered genotyping that had insignificant statistical association with the severity of COVID-19

ACE2 Single nucleotide variant	Genotype	Severity		P	OR (CI 95%)	FDR^b^
		**Serious** **n = 68**	**Non-Serious** **n = 56**			
rs1391451327	AA GG + TT	61 (0.897) 7 (0.103)	55 (0.982) 1 (0.018)	0.090	0.158 (0.19–1.329)	0.54
rs2285666	CC AA + TT + CT	32 (0.47) 36 (0.53)	29 (0.517) 27 (0.483)	0.600	0.828 (0.408–1.680)	1.80
rs768883316	TT CC + AA	64 0.941) 4 (0.059)	53 (0.946) 3 (0.054)	0.900	0.906 (0.194–4.227)	1.35
rs180878567	AA TT + GG	68 (1.00) 0 (0)	53 (0.946) 3 (0.054)	0.999	NA	1.19
rs756597390	AA CC + TT	67 (0.985) 1 (0.015)	55 (0.982) 1 (0.018)	0.890	1.218 (0.074–19.92)	1.78
rs768583671	AA CC + GG + TT	65 (0.955) 3 (0.045)	56 (1.00) 0 (0)	0.999	NA	0.999

^b^Benjamini-Hockberg method. CI, confidence Interval; FDR, false discovery rate; NA, does not apply; OR, odds ratio

Additionally, we aimed to determine the correlation between all 22 ACE2 variants and various factors including ethnicity, age groups, comorbidities, and gender in our current study. However, none of the variants displayed any statistical significance when applying the regression model with ethnicity, except for rs2285666, which showed significant statistical linkage with gender (p-value 0.034), and rs768883316, which had a significant statistical association with age groups (p-value 0.026). This information can be found in Tables 5 and 6.

**Table 5:** Genotype distribution in COVID-19 severity groups disaggregated by age group. rs76888331 was found to have positive statistical association with the age having the 95 patients with age > 50 years, serious (53 patients) with TT genotyping (53 patients)

ACE2 Single nucleotide variant	Age Group	Severity	Genotype frequency			P	OR (CI 95%)
			**CC**	**AA**	**TT**		
rs768883316	< =50 Years (n = 29) > 50 Years (n = 95)	Serious (n = 15) Non serious (n = 14) Serious (n = 53) Non serious (n = 42)	0 (0) 0 (0) 0 (0) 2 (0.048)	4 (0.266) 0 (0) 0 (0) 1 (0.024)	11 (0.734) 14 (1.00) 53 (1.00) 39 (0.928)	0.026	1.953 (1.085–3.514)

CI, confidence Interval; NA, does not apply; OR, odds ratio

**Table 6:** Genotype distribution in COVID-19 severity groups disaggregated by age gender. rs2285666 had the positive association with gender with male dominance (69 patients), having the CC genotyping (21 patients)

ACE2 Single nucleotide variant	Gender	Severity	Genotype frequency				P	OR (CI 95%)
			**CC**	**AA**	CT	TT		
rs2285666	Male (n = 69) Female (n = 55)	Serious (n = 38) Non serious (n = 31) Serious (n = 30) Non serious (n = 25)	21 (0.553) 19 (0.613) 11 (0.367) 10 (0.4)	0 (0) 0 (0) 1 (0.034) 0 (0)	0 (0) 1 (0.032) 2 (0.066) 3 (0.12)	17 (0.447) 11 (0.355) 16 (0.533) 12 (0.48)	0.034	1.438 (1.028–2.011)

CI, confidence Interval; FDR, false discovery rate; NA, does not apply; OR, odds ratio

Haplotypes ATC of three polymorphisms (rs560997634, rs201159862 and rs751170930) commonly found in 120 (69.77%) patients including the 54; 43.54% non-serious and 66; 53.22% serious and TTTGTAGTTAGTA haplotype consisting of 13 polymorphisms (rs756737634, rs146991645, rs1601703288, rs1927830489, rs1927831624, rs764947941, rs752242172, rs73195521, rs781378335, rs756597390, rs780478736, rs148006212, rs768583671) in 112 (90.32%) with non-serious 48; 38.7%, serious 64; 51.61% had significance statistical association with the severity having p = value 0.029 and 0.001 respectively. Table: 7.

**Table 7:** Frequency of ACE2 Haplotypes distribution in COVID-19 patients: ATC and TTTGTAGTTAGTA were most common haplotypes found. ATC haplotypes found in 120 patients having 66 serious patients while TTTGTAGTTAGTA found in 112 patients with 64 serious patients

Haplotype	Frequency	Severity	OR (95%: CI)	p-value
ATC	120 (69.77%)	Non-Serious (54; 43.54%) Serious (66; 53.22%)	3.27 (1.10–9.70)	0.029*
TTTGTAGTTAGTA	112 (90.3%)	Non-Serious (48; 38.70%) Serious (64; 51.61%)	5.44 (2.12–13.93)	< 0.001*

OR, odds ratio; CI, confidence interval; *P < 0.05 was considered statistically significant

## Discussion

The Coronavirus is a diverse group of viruses that can lead to common colds, as well as severe respiratory illnesses such as pneumonia, and even conditions like SARS and Middle East Respiratory Syndrome (MERS) [10]. There has been limited publication on prognostic variables for COVID-19. Additionally, the investigation has noted a predominance of males, potentially attributable to the localization of the ACE2 gene on the X-chromosome [11]. The prevalent hypothesis suggests that COVID-19 infection rates are higher among males compared to females. Recent research, conducted through a case-control study on a Chinese demographic, reinforces this theory. This study discovered an inverse correlation between ACE2 expression levels and estrogen levels, suggesting that estrogen may contribute to the suppression of ACE2 expression. This indicates that females may possess a protective factor against COVID-19 infection compared to their male counterparts, possibly due to the influence of estrogen on ACE2 expression [12].

The analysis shows that COVID-19 tends to be more severe among individuals over 50 years old. This age group was found to have a higher incidence of severe cases in the study [13]. However, it is currently unknown how many ACE2 receptors are present in the organs of elderly individuals who are at a higher risk of severe illness. Additionally, the immune system tends to weaken with age, and comorbidities like diabetes, hypertension, and others can further impact immune system deterioration, increasing the risk of contracting COVID-19 infection [14]. ACE inhibitors and ARBs are used to treat hypertension, which causes ACE2 to be unregulated. These findings suggest that ACE2 expression is elevated in diabetes, and that therapy with ACE inhibitors and ARBs improves ACE2 expression [15]. COVID-19 infection would be aided by increased ACE2 expression. Diabetes, cerebral stroke, and hypertension have all been linked to ACE2 polymorphisms, particularly in Asian populations. An individual’s sensitivity may be affected by a combination of therapy and the ACE2 polymorphism [16].

The severity of COVID-19 disease may also vary geographically, as rural hospitals and communities sometimes lack services. Ethnicity is influenced by genetic backgrounds, environmental factors, as well as cultural and behavioral norms [17]. Therefore, it is conceivable that these societal factors could influence the severity of COVID-19 disease in populations of people of color [18]. Although it was highlighted that there was conflicting information about the link between ethnicity and mortality, there was consistent evidence that ethnic minorities had higher infection rates. Different people have different cellular amounts of *ACE2* expression [19].

SARS-CoV-2 susceptibility and COVID-19 illness outcome could all be influenced by ACE2 gene polymorphism, human ACE2 mRNA expression, and human ACE2 protein polymorphism [20]. Human ACE2 has been discovered as a host cell receptor responsible for mediating coronavirus infection in studies on host-pathogen interaction (COVID-19). Srivastava et al., in his report found a significant difference in alleles between Europeans and Asians for the ACE2 polymorphism rs2285666. The alternative allele (TT-plus strand or AA-minus strand) of rs2285666 has been found to increase the expression level of this gene by up to 50%, suggesting that it may have a role in SARS-CoV-2 susceptibility [21]. Because rs2285666 has been linked to hypertension, type 2 diabetes, and coronary artery disease, it could be a predisposing factor for the comorbidities seen in COVID-19 individuals. The variant rs2285666 is found near the start of intron 3, and it could potentially affect gene expression and protein levels by alternative splicing mechanisms [22]. Some genetic variants in the *ACE2* can bring about variations in binding affinity of ACE-2 for SARS COV-2 RBD. rs2285666 is one of these SNPs whose wild type enhances *ACE2* production with a greater affinity for SARS-CoV-2. Wooster et al. identified that six SNPs in the *ACE2* gene region that increase the expression level of *ACE2* receptors are significantly associated with a higher risk of hospitalization in patients with COVID-19 [23].

Based on the findings of our current study, we discovered that the common SNPs associated with rs2285666 in COVID-19-positive patients were as follows: 55.9% CC, 39.2% TT, 4.2% CT heterozygosity, and 0.7% AA genotypic carrier. These findings align with the percentages projected for the Pakistani population (PJL: Punjabi Lahore) database, which are 59.7% for CC, 40.3% for TT, and 2.7% for CT heterozygosity. We observed CT heterozygosity in six patients, including one asymptomatic, three mild, one severe, and one critical patient. These results are also supported by the NCBI thousand genome project.

This study also demonstrated that the ACE2 rs2285666 CC genotype had increased risk to develop the COVID-19 disease as compared to ACE2 rs2285666 TT genotype [24]. In a study evaluating the association between rs2285666 genotypes and circulating ACE2 in DM patients, it was discovered that the AA genotype has the highest level of expression compared to the other genotypes [25]. The wild genotype and the C allele was substantially related with the prevalence and risk of SARS-CoV-2 infection in our investigation, comparable to the findings in Indian and Caucasian populations [24]. As a result, it is hypothesized that these functional ACE2 variations could influence disease progression. The A-allele frequency was much greater in patients in our study, although it was linked to COVID-19. Given that ACE2 receptor gene expression may influence an individual’s susceptibility to infection, we hypothesize those genetic variations in the noncoding regions of the ACE2 receptor gene or other noncoding DNAs that regulate ACE gene expression levels may play a role in the severity of the disease [26].

A German study showed that G allele and GG genotype of rs2285666 were linked to COVID-19 vulnerability, especially in critically ill individuals. That demonstrated the susceptibility to COVID-19, particularly in seriously ill patients [27]. This observation was also advocated by other studies, the GG genotype of the ACE2 rs2285666 (G8790A) polymorphism was previously linked to a 50% reduction in protein production when compared to the AA genotype [28]. As shown, the correlation of the rs2285666 polymorphisms with COVID-19 susceptibility varies between studies around the world, which could be due to ethnic differences in populations, as these variants show some population-based differences. Given that ACE2 rs2285666 has been linked to hypertension, this polymorphism may influence susceptibility to SARS-CoV-2 infection and the severity of COVID-19. Reduced ACE2 protein levels and the loss of the protective impact of the ACE2/MAS pathway both contributed to the severe effects of SARS-CoV-2 infection, supporting this hypothesis [28]. Following up on the previous point, Möhlendick et al. found that the ACE2 rs2285666 polymorphism caused function loss in 297 COVID-19 positive people. They also discovered that the ACE2 rs2285666 GG genotype or G allele was linked to a two-fold greater risk of infection and a three-fold increased risk of severe disease or fatality [28]. Celik et al., on the other hand, found no link between this polymorphism and the severity of COVID-19. This could be related to epigenetic mechanisms that control ACE2 receptor expression, as well as alterations in other genes such as pro-inflammatory cytokines and coagulation indicators, all of which can affect a patient’s prognosis [29]. ATC and TTTGTAGTTAGTA haplotypes had more association with the risk of COVID-19 than other haplotypes. These haplotypes were profound in serious patients with severe and critical cases. Currently we did not find any study that highlighted the ACE2 haplotypes to compare our results.

In this study, the haplotypes ATC and TTTGTAGTTAGTA were analyzed. The former is composed of three polymorphisms (rs560997634, rs201159862, and rs751170930), and the latter is composed of 13 polymorphisms (rs756737634-rs768583671). The results showed that these haplotypes had a statistically significant association with the severity of the condition, with p-values of 0.029 and 0.001, respectively. Most of the affected patients required intensive care, which may be attributed to the different haplotypes found in different populations, affecting the body’s immune system, expression levels, and protein structures in varied ways [30]. Each population has unique genetic makeup, influenced by environmental factors and risk exposure, which can result in variations in gene expression and mutations. The impact of mutations within a gene may differ depending on their location on the same chromosome, a haplotype, or opposite homologous chromosomes [31]. In haplotype studies, it is common to analyze multiple closely linked markers as they offer more valuable information compared to a single marker. This may be a significant factor in understanding the severity of COVID-19 in the current study. The immune response’s effectiveness in producing a specific antiviral immunity without damaging the host tissues depends on various environmental and genetic factors, which are also applicable to the present findings [32].

In the current study, it was observed that several patients with severe diseases possessed SNPs at the slicing donor site. These sites hold significant immune regulatory functions in the body. During the splicing process, the transcript eliminates intronic sequences, leaving behind only the coding sequences in mature mRNA [33]. It is crucial to avoid errors during the splicing process since they can result in improper intron removal and alter the open reading frame. Overall, the splicing process is a complex event that plays a vital role in protein synthesis. Any changes in this process can reduce the messenger RNA level, leading to a lack of protein and causing abnormal cellular metabolism or function. The SNPs discovered in this study may impact protein function, structure, expression levels, and binding affinities, leading to different clinical outcomes and presentations [11]. Furthermore, dysregulation and significantly differential expression of transcripts due to the SNPs at the splice donor site are linked to many viral diseases and COVID-19 severity [34].

To the best of our knowledge, based on a comprehensive review of existing literature, this study is the first of its kind to focus on the distinct ACE2 variants related to disease severity, specifically using human samples from a subset of the Pakistani population. While the single nucleotide polymorphisms (SNPs) identified in our study were associated with viral pathogenesis, no evidence was found linking these genetic variations to an increased susceptibility to COVID-19. This could potentially be due to limited evidence and data scarcity, indicating a need for further investigation with larger sample sizes. Apart from genetic predispositions, numerous factors like age, gender, ethnicity, and co-existing health conditions, are recognized to influence a population’s vulnerability to SARS-CoV-2 infection. However, it is crucial to exercise caution in the interpretation of our findings due to the nature of our study. Since it was small and selective, the results may not be generalizable to a larger population or to communities of diverse nationalities. For our hypotheses to gain broader acceptance, they must be substantiated through additional independent research.

## Conclusion

A statistically significant predominance of males with severe cases of COVID-19 was observed, with the older age group being primarily affected. As disease severity increased, there was a concurrent sequential escalation in the levels of pertinent inflammatory markers. Certain ACE2 polymorphisms, particularly ACE2 rs781378335, and rs2285666, emerged as independent associations with the severity of COVID-19. Specifically, the ACE2 rs2768883316 polymorphism appears to correlate with a heightened risk of contracting COVID-19 in later life stages. Moreover, male susceptibility to severe manifestations of the disease was particularly associated with the ACE2 rs2285666 polymorphism.

## Electronic supplementary material

Below is the link to the electronic supplementary material.

**Supplementary Figure: 1** (a): Gel doc for *ACE2* demonstrating the bands for PCR products.

**Supplementary Table 1**: *ACE2* haplotypes, haplotype frequency and permutation test for all haplotypes organized in block 1 and block 2
