# Supplementary material for: Angiotensin-converting enzyme 2 (ACE2) polymorphisms and susceptibility of severe SARS-CoV-2 in a subset of Pakistani population
Source: Virol J. 2023 Jun 12;20:120. doi: 10.1186/s12985-023-02091-2 (PMC10258755; doi:10.1186/s12985-023-02091-2)
Supplement: Supplementary file 1 — Supplementary Figure: 1 (a): Gel doc for ACE2 demonstrating the bands for PCR products. [file 12985_2023_2091_MOESM1_ESM.docx]

Supplementary Figure: 1 (a): Gel doc for *ACE2* demonstrating the bands for PCR products


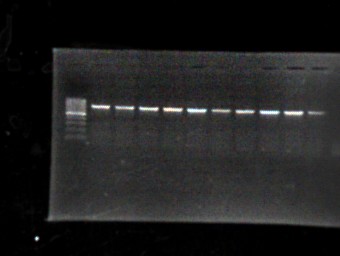


L ZU75 ZU76 ZU77 ZU78 ZU79 ZU80 ZU81 ZU 82 ZU83 ZU84

800bp
