# Supplementary material for: Angiotensin-converting enzyme 2 (ACE2) polymorphisms and susceptibility of severe SARS-CoV-2 in a subset of Pakistani population
Source: Virol J. 2023 Jun 12;20:120. doi: 10.1186/s12985-023-02091-2 (PMC10258755; doi:10.1186/s12985-023-02091-2)
Supplement: Supplementary file 2 — Supplementary Table 1: ACE2 haplotypes, haplotype frequency and permutation test for all haplotypes organized in block 1 and block 2 [file 12985_2023_2091_MOESM2_ESM.docx]

Supplementary Table 1: *ACE2* haplotypes, haplotype frequency and permutation test for all haplotypes organized in block 1 and block 2

| Block 01 | Haplotype | Frequency | Permutation p-value |
| --- | --- | --- | --- |
|  | ATC | 0.958 | 1.00 |
|  | AAC | 0.024 | 1.00 |
|  | ATG | 0.012 | 1.00 |
| Block 02 | TTTGTAGTTAGTA | 0.594 | 1.00 |
|  | TTTGTAGTTAATA | 0.327 | 1.00 |
|  | TGTGTAGTTAGTA | 0.018 | 1.00 |
|  | TTTGTAGTCAGTA | 0.018 | 1.00 |
|  | TTAGTAGTTAGTA | 0.012 | 1.00 |
|  | GTTGTTGGTCAGA | 0.012 | 1.00 |
